# Serum kisspeptin as a promising biomarker for PCOS: a mini review of current evidence and future prospects

**DOI:** 10.1186/s40842-024-00190-9

**Published:** 2024-09-30

**Authors:** Emmanuel Kokori, Gbolahan Olatunji, Rosemary Komolafe, Ikponmwosa Jude Ogieuhi, Bonaventure Ukoaka, Irene Ajayi, Nicholas Aderinto

**Affiliations:** 1 Department of Medicine and Surgery, University of Ilorin, Ilorin, Nigeria; 2 Siberian State Medical University, Tomsk, Russia; 3 Department of Internal Medicine, Asokoro District Hospital, Abuja, Nigeria; 4 Department of Medicine and Surgery, Ladoke Akintola University of Technology, Old Oyo/ Ilorin Rd, P.M.B 4000, Ogbomosho, Oyo-State 210214 Nigeria

**Keywords:** Polycystic ovary syndrome (PCOS), Kisspeptin, Biomarker, Hypothalamic-pituitary-gonadal (HPG) axis

## Abstract

Polycystic ovary syndrome (PCOS) is a prevalent endocrine disorder affecting women of reproductive age, characterised by its multifactorial nature and intricate interplay of genetic, hormonal, and environmental factors. As the search for reliable biomarkers intensifies, serum kisspeptin emerges as a promising candidate due to its central role in regulating the hypothalamic-pituitary-gonadal (HPG) axis. This review aims to consolidate the evolving understanding of kisspeptin as a potential PCOS biomarker, comprehensively exploring its physiological basis, diagnostic challenges in PCOS, and clinical implications. Diagnostic challenges in PCOS are addressed, underscoring the limitations of current criteria and the need for objective and standardised biomarkers. Kisspeptin’s introduction as a potential biomarker brings forth both promises and challenges in terms of its diagnostic utility. The review recognises the importance of standardisation in research methodologies and emphasises the exploration of genetic polymorphisms to enhance kisspeptin’s robustness as a diagnostic tool.

## Introduction

Polycystic ovary syndrome (PCOS) is a prevalent endocrine disorder among women of reproductive age, characterised by its complexity, familial nature, and polygenetic metabolic condition. It affects 5 to 15% of the population, with the prevalence dependent on the diagnostic criteria utilised [1, 2]. Irregular menstrual cycles, hyperandrogenism (including hirsutism, acne, and alopecia), and polycystic ovaries on ultrasonography differentiate it. Obesity, insulin resistance, and infertility are typical additional features [1]. Although the exact cause of this syndrome is still largely unknown, accumulating evidence indicates that PCOS could be a multifactorial disorder influenced significantly by epigenetic and environmental factors, such as lifestyle choices and diet [3–6]. PCOS poses significant challenges in diagnosis and management, impacting the overall health and fertility of affected individuals [7, 8]. The interplay of genetic, hormonal, and environmental factors complicates our understanding of PCOS [9].

Biomarker usage in PCOS is advancing rapidly [10]. While PCOS is a well-recognized and prevalent condition, searching for reliable biomarkers remains a priority for enhancing early detection and targeted management [11]. Due to its intimate involvement in reproductive physiology, Serum kisspeptin presents a compelling candidate for such a role [12]. Preliminary studies have suggested alterations in serum kisspeptin levels in women with PCOS, sparking interest in its potential as a diagnostic biomarker [13, 14]. Within this dynamic landscape, kisspeptin, a neuropeptide with a crucial role in regulating the hypothalamic-pituitary-gonadal (HPG) axis, is emerging as a promising biomarker for PCOS [15]. Kisspeptin’s involvement in controlling gonadotropin-releasing hormone (GnRH) secretion positions it as a pivotal factor in the reproductive dysfunction observed in PCOS [16].

Exploring the relationship between serum kisspeptin levels and PCOS can enhance understanding of the disorder and promise improved precision in diagnostics. Amid ongoing advancements, this review aims to consolidate the evolving knowledge of kisspeptin as a biomarker in PCOS, contributing to a broader understanding of this challenging condition.

## Methodology

This review explored serum kisspeptin’s current status as a potential PCOS biomarker. It searched electronic databases, including PubMed, Scopus, and Web of Science. The search strategy involved using relevant keywords, such as “kisspeptin,” “PCOS,” and “biomarker,” with Boolean operators to refine and broaden the scope of the search. The review considered articles published up until December 2023 to encompass the most recent developments in the field.

Studies were included if they specifically explored the relationship between serum kisspeptin levels and PCOS. Exclusion criteria were applied to filter out studies not written in English, those exclusive to animal models, and those lacking clear relevance to the biomarker aspect of PCOS. Initial screening involved evaluating titles and abstracts to determine relevance. Subsequently, a detailed full-text screening was performed based on predetermined inclusion and exclusion criteria. This process ensured that studies that met the defined criteria for in-depth analysis were selected.

Findings from the selected studies were synthesised to offer an overview of the existing evidence. This synthesis included an analysis of patterns, consistencies, and discrepancies in the results, contributing to understanding the potential role of serum kisspeptin as a biomarker in PCOS.

### Current diagnostic challenges in PCOS

PCOS presents a diagnostic challenge due to its diverse features, prompting the proposal of various diagnostic criteria since 1935, when Stein and Leventhal introduced the first criterion [17]. An optimal criterion balances specificity and sensitivity, which is crucial for identifying affected individuals and ruling out others [18]. PCOS, a common endocrine disorder affecting women of reproductive age [19], requires a combination of clinical, biochemical, and imaging criteria for an accurate diagnosis. The Rotterdam Criteria 2003 [20] demand at least 2 of 3 criteria: anovulation/oligoovulation, clinical or biochemical evidence of hyperandrogenism, and polycystic ovaries on ultrasound. The Androgen Excess Society (AES) in 2006 prioritised hyperandrogenism but acknowledged the importance of ovarian morphology [21]. Current recommendations emphasise evaluating biochemical hyperandrogenism through total free testosterone and clinical hyperandrogenism using the modified Ferriman-Gallwey score [18]. Diagnosis includes ovulatory dysfunction assessment, and polycystic ovarian morphology (PCOM) is defined as more than 20 follicles per ovary and/or an ovarian volume exceeding 10 cubic cm.

The complexity of PCOS, its heterogeneous clinical presentation, and the need to rule out other endocrine pathologies contribute to diagnostic challenges [22]. PCOS, often diagnosed in adolescence, may not be recognised until infertility becomes apparent [23–27]. Overlapping syndromes with other diseases, like thyroid disorders, can lead to delayed diagnosis and misclassification [28]. In addition, ethnicity and geographical variation contribute to the heterogeneity in PCOS presentations [29]. Clinical evidence suggests metabolic manifestations are more prevalent in America and Asia, while hyperandrogenism is more common in the Middle East and Europe [30]. Inconsistencies in diagnosis arise from the subjective nature of symptoms [31]. Diagnostic limitations include the reliance on menstrual history, particularly in individuals using hormonal contraceptives, which mask irregularities [32]. Hirsutism’s subjective nature and varying prevalence among populations pose challenges [33]. Ovarian morphology criteria often lack specificity, as age, BMI, and ethnicity influence ultrasound findings [34].

Given these challenges, the quest for reliable biomarkers is essential for accurate and efficient PCOS diagnosis. The heterogeneous nature of PCOS necessitates biomarkers offering an objective and standardised approach to categorising all phenotypes [35]. Such markers can enhance diagnostic accuracy, decrease misdiagnosis rates, and guide individualised treatments. Biomarkers’ role extends to monitoring treatment efficacy, aiding early diagnosis, and intervening to prevent long-term complications. Reliable biomarkers can reduce variability in PCOS diagnosis based on subjective symptoms, contributing to more consistent case identification. This objectivity becomes crucial in research and clinical trials, providing measurable outcomes and aiding in developing improved treatments. The ongoing studies on novel biomarkers promise a future where these markers contribute to precise PCOS classification, reducing ambiguity and advancing personalised approaches to diagnosis and management within precision medicine [36, 37].

### Physiological basis of kisspeptin

The hypothalamic-pituitary-gonadal (HPG) axis is a central component in reproduction [38]. Comprising the gonads, anterior hypothalamus, and pituitary, this system is pivotal in modulating animal and human reproductive processes [39]. Key aspects, such as spermatogenesis in males and the processes of follicular development, egg maturation, and ovulation in females, are significantly influenced by the HPG axis [40, 41].

The neuropeptide kisspeptin, first identified by Lee et al. [42], operates through the GPR54 G-protein-coupled receptor [43]. Originally linked to cancer metastasis, further exploration has established its profound connection with reproduction [43]. By modulating GnRH secretion, kisspeptin emerges as a critical regulator within the HPG axis [44, 45]. Recent studies identify kisspeptin neurons in the arcuate nucleus (ARC), co-expressing dynorphin and neurokinin B (NKB), collectively known as KNDy neurons [46, 47]. These neurons are central to positive and negative oestrogen feedback mechanisms on GnRH secretion [46]. Through the HPG axis, kisspeptin governs female follicle development, oocyte maturation, and ovulation, contributing significantly to the initiation of puberty [45]. In male reproduction, kisspeptin regulates spermatogenesis, Leydig cells, sperm functions, and reproductive behaviours [46].

The release of kisspeptin from the hypothalamus, influenced by various regulatory factors, modulates GnRH secretion from GnRH neurons, effectively managing the reproductive axis [47]. Binding to its receptor, kisspeptin receptor (KISS1R), stimulates GnRH secretion, thus regulating the hypothalamic-pituitary-gonadal axis (HPG) [46]. Studies confirm that administering exogenous kisspeptin induces a reproductive cascade in animals and humans [48, 49]. This effect is inhibited by a GnRH antagonist, reinforcing kisspeptin’s preeminent position along the HPG axis. Modern techniques, including optogenetics, demonstrate that the synchronous activation of ARC kisspeptin neurons induces pulsatile luteinising hormone (LH) release in rodents, dependent on sex steroids [50, 51].

In PCOS, characterised by HPG axis disturbance, kisspeptin, encoded by the KISS1 gene, stimulates gonadotropin secretion, releasing LH and follicle-stimulating hormone (FSH) [51]. Interactions with its receptors, KISS1R/GPR54, are essential for HPG axis regulation [50]. Polymorphisms or mutations in the KISS1 gene disrupt the HPG axis, influencing the kisspeptin signalling pathway [52]. Such deviations lead to atypical GnRH pulse secretion, elevating the LH/FSH ratio, impacting androgen levels, and affecting ovulation. Elevated androgen concentrations exacerbate PCOS symptoms [53].

Various single nucleotide polymorphisms (SNPs) in the KISS1 gene have been identified as disruptors of the reproductive system via the HPG axis, playing a crucial role in PCOS pathogenesis [54]. These mutations can modify kisspeptin’s structure, function, and binding affinity to its receptor, GPR54/KISS1R [55]. As a result, polymorphism-induced anomalous gonadotropin-releasing hormone (GnRH) secretion leads to increased LH and androgen synthesis (estradiol and testosterone) [53]. Tang et al. (2019) revealed elevated circulating kisspeptin levels among women with PCOS, supporting the hypothesis that an overactive KISS1 system could contribute to syndrome development [54]. This heightened HPG-axis activity leads to irregular menstrual cycles and excessive androgen secretion [54].

Hypotheses suggest that elevated steroid hormones, influencing the GnRH and HPG axes via the kisspeptin-GPR54 signalling pathway, may contribute to PCOS development [55]. Increased testosterone inhibits GnRH, leading to PCOS symptoms such as male pattern baldness, hirsutism, and acne vulgaris [55, 56]. A continuous increase in LH pulsatility lowers FSH levels, causing anovulation and follicle stimulation cessation, ultimately resulting in polycystic ovaries [57]. These findings support the notion that an overactive KISS1 system could contribute to PCOS development, characterised by irregular menstrual cycles, excessive androgen secretion, and an activated HPG-axis [53].

### Kisspeptin as a potential biomarker for PCOS

Recent studies have explored the relationship between kisspeptin and PCOS. Akad et al. [37] conducted a prospective case study, revealing significantly higher kisspeptin levels in PCOS patients with primary or secondary infertility during the follicular phase of the menstrual cycle. This elevation suggests a potential link between KISS1 overstimulation, hyperstimulation of the HPG axis, irregular menstrual cycles, and increased testosterone secretion. However, findings regarding kisspeptin levels in PCOS are varied. Some studies reported increased levels [58, 59], while others showed a negative correlation [60, 61]. Notably, kisspeptin’s involvement in regulating the HPG axis makes a positive correlation with LH plausible [62]. Ozlen et al. [63] found slightly elevated plasma kisspeptin levels in women with PCOS, positively correlated with LH and leptin levels.

Studies among diverse populations, such as the Sri Lankan study by Umayal et al. [64], reinforced the association between higher kisspeptin levels and PCOS. Meixiu et al. [65] explored kisspeptin’s correlation with biochemical markers in obese and non-obese women with PCOS, suggesting its potential role in treatment, prognostication, and clinical evaluation. While serum kisspeptin’s sensitivity and specificity as a PCOS biomarker are not extensively explored, Yilmaz et al. [66] and Cihan et al. [67] provided cutoff values with promising specificity and sensitivity. However, more research is needed to understand its diagnostic utility comprehensively.

Comparing kisspeptin with established biomarkers like anti-Mullerian hormone (AMH), sex hormone-binding globulin (SHBG), CpG methylation biomarkers, and serum androgens can shed light on its diagnostic potential and interactions with other hormonal aspects of PCOS. Additionally, assessing kisspeptin levels in relation to chronic inflammatory markers may provide insight into the inflammatory aspect of PCOS and its potential cardiovascular and metabolic implications.

### Clinical implications, diagnostic utility, and challenges

While this review provides evidence linking elevated kisspeptin expression to PCOS pathology, its status as a diagnostic biomarker remains unestablished. However, early detection of heightened kisspeptin levels could offer valuable clinical assumptions, potentially facilitating earlier diagnosis and intervention. The complex and heterogeneous nature of PCOS symptoms, varying kisspeptin levels among phenotypic variants, and the association of kisspeptin with specific PCOS symptoms highlight its potential role in identifying at-risk individuals for further investigation and early diagnosis [67].

Moving beyond diagnosis, kisspeptin holds promise for influencing ovulation disorders and energy metabolism in PCOS patients. Current management strategies, albeit potent, are associated with severe complications such as ovarian hyperstimulation syndrome (OHSS) [67]. Studies exploring the stimulatory effects of kisspeptin on the HPG axis for endogenous gonadotrophin release present a potential avenue for novel therapies [68, 69]. Preliminary studies suggest its effectiveness in triggering oocyte maturation in females undergoing in vitro fertilisation (IVF) [39, 53].

Despite these promising advancements, kisspeptin remains underutilised both as a diagnostic tool and as a treatment modality for PCOS-induced fertility. Ongoing studies exploring the potential of kisspeptin antagonists and the development of rapid diagnostic tests present exciting prospects for improving PCOS management and diagnosis [70, 71]. Furthermore, the application of innovative technologies like nanopeptamers and Inductively Coupled Plasma-Mass Spectrometry (ICP-MS) in point-of-care testing underscores the evolving landscape of diagnostic modalities for kisspeptin [72, 73].

The challenges in kisspeptin research are acknowledged, including variations in study types, designs, and demographic factors contributing to inconsistencies in findings. Standardisation of factors such as BMI cutoffs and detailed descriptions of sample collection procedures are crucial for accurate and comparable data. The multifaceted nature of PCOS, influenced by genomic variations and environmental factors, adds complexity to interpreting kisspeptin levels, urging the need for further research into genetic polymorphisms of KISS1.

## Conclusion

PCOS is a complex endocrine disorder affecting women’s reproductive years, involving genetic, hormonal, and environmental factors. The search for reliable biomarkers is gaining momentum to enhance early detection and guide tailored interventions. Serum kisspeptin, a neuropeptide involved in regulating the HPG axis, is a promising candidate for a PCOS biomarker. The review highlights the need for objective and standardised approaches to address the diagnostic challenges in PCOS. Kisspeptin introduces a new dimension in PCOS diagnostics, promising more precise categorization and personalised treatments. However, the clinical implications, diagnostic utility, and challenges associated with kisspeptin present both the promises and hurdles in its journey towards becoming a reliable PCOS biomarker. The review acknowledges the need for standardisation in research methodologies and exploration of genetic polymorphisms to enhance the robustness of kisspeptin as a diagnostic tool.

## Data Availability

No new datasets were generated for this study. All data used are within this manuscript.

## Abbreviations

PCOS: Polycystic Ovary Syndrome
BMI: Body Mass Index
GnRH: Gonadotropin-Releasing Hormone
HPG: Hypothalamic-Pituitary-Gonadal
IVF: In Vitro Fertilization
OHSS: Ovarian Hyperstimulation Syndrome
AMH: Anti-Mullerian Hormone
SHBG: Sex Hormone-Binding Globulin
CpG: Cytosine-phosphate-Guanine
SNPs: Single Nucleotide Polymorphisms
